# SELF-PERCEPTIONS OF AGING AMONG VERY OLD PARENTS AND THEIR OLD CHILDREN IN KOREA

**DOI:** 10.1093/geroni/igad104.0920

**Published:** 2023-12-21

**Authors:** Kyungmin Kim, Joohyun Kim, Joohong Min, Yijung Kim, Kathrin Boerner, Daniela Jopp, Gyounghae Han

**Affiliations:** Seoul National University, Seoul, Republic of Korea; Seoul National University, Seoul, Republic of Korea; Jeju National University, Jeju, Republic of Korea; The University of Texas at Austin, Austin, Texas, United States; University of Massachusetts Boston, Boston, Massachusetts, United States; University of Lausanne, Lausanne, Vaud, Switzerland; Seoul National University, Seoul, Republic of Korea

## Abstract

Research has documented the health consequences of how individuals view their own age-related changes and aging (i.e., self-perceptions of aging) in later life. However, less is known about how individuals’ self-perceptions of aging are shaped by their intergenerational experiences. This study aims to examine very old parents’ and their old children’s self-perceptions of aging within the dyads—where parents and children age together. Utilizing data from 105 dyads of parents (aged 81+) and their children (aged 65+) in South Korea, we examined how parents’ and children’s health status and care experiences are associated with their self-perceptions on aging. On average, parents reported less positive self-perceptions of aging than their children; parents’ self-perceptions of aging were positively correlated with their child’s self-perceptions of aging. A path model found that care experiences within the parent-child dyads were significantly associated with self-perceptions of aging for both parents and children—such that more caregiving burden was associated with less positive self-perceptions of aging for children and higher levels of satisfaction with received care from the child were associated with more positive self-perceptions of aging for parents. However, health status was significant only for parents; that is, parents with fewer functional limitations in activities of daily living tended to show more positive self-perceptions of aging. The findings highlight the interdependence of self-perceptions of aging within the parent-child dyads in very late life, suggesting that care experiences within the prolonged relationship constellation should be considered to enhance older adults’ views on age-related gains and loss.

